# Kaposi’s sarcoma-associated herpesvirus G-protein coupled receptor activates the canonical Wnt/β-catenin signaling pathway

**DOI:** 10.1186/s12985-014-0218-8

**Published:** 2014-12-17

**Authors:** Magdalena Angelova, MaryBeth Ferris, Kenneth F Swan, Harris E McFerrin, Gabriella Pridjian, Cindy A Morris, Deborah E Sullivan

**Affiliations:** Department of Microbiology and Immunology, Tulane University School of Medicine, 1430 Tulane Avenue, New Orleans, LA USA; Department of Obstetrics and Gynecology, Tulane University School of Medicine, 1430 Tulane Avenue, New Orleans, LA USA; Biology Department, Xavier University, 1 Drexel Drive, New Orleans, LA USA

**Keywords:** Kaposi’s sarcoma-associated herpesvirus, Wnt/β-catenin signaling, Angiogenesis

## Abstract

**Background:**

KSHV is a tumorigenic γ-herpesvirus that has been identified as the etiologic agent of Kaposi’s sarcoma (KS), a multifocal highly vascularized neoplasm that is the most common malignancy associated with acquired immunodeficiency syndrome (AIDS). The virus encodes a constitutively active chemokine receptor homologue, vGPCR that possesses potent angiogenic and tumorigenic properties, and is critical for KSHV pathobiology. To date, a number of signaling pathways have been identified as key in mediating vGPCR oncogenic potential.

**Findings:**

In this study, we identify a novel pathway, the Wnt/β-catenin pathway, which is dysregulated by vGPCR expression in endothelial cells. Expression of vGPCR in endothelial cells enhances the nuclear accumulation of β-catenin, that correlates with an increase in β-catenin transcriptional activity. Activation of β-catenin signaling by vGPCR is dependent on the PI3K/Akt pathway, as treatment of vGPCR-expressing cells with a pharmacological inhibitor of PI3K, leads to a decreased activation of a β-catenin-driven reporter, a significant decrease in expression of β-catenin target genes, and reduced endothelial tube formation.

**Conclusions:**

Given the critical role of Wnt/β-catenin signaling in angiogenesis and tumorigenesis, the findings from this study suggest a novel mechanism in KSHV-induced malignancies.

**Electronic supplementary material:**

The online version of this article (doi:10.1186/s12985-014-0218-8) contains supplementary material, which is available to authorized users.

## Background

Kaposi’s sarcoma-associated herpesvirus (KSHV) is a tumorigenic gammaherpesvirus that is the etiologic agent of Kaposi’s sarcoma (KS). KS is a multifocal, highly vascular tumor that develops on the lower extremities, mucous membranes or internal organs. Co-infection with HIV markedly increases the risk of KS development [1]. Although highly active anti-retroviral therapy (HAART) has decreased the incidence of AIDS-related KS, it remains an incurable tumor for which there is no established treatment [2]. KS tumors are characterized by highly proliferating, elongated, spindle shaped cells of endothelial origin. The majority of cells in the tumor are latently infected with KSHV, with less than 3% of the cells expressing viral lytic proteins [3]. It has been proposed that dysregulation of the viral gene program leads to nonlytic expression of a virally-encoded G-protein coupled receptor (vGPCR) homologue [4]. vGPCR has potent angiogenic and tumorigenic properties and its transgenic expression *in vivo* is sufficient to induce the development of angioproliferative lesions characterized by prominent inflammation, dysregulated angiogenesis and the presence of endothelial cells with a spindle morphology [5]. Consequently, vGPCR has become a potential therapeutic target for KS.

The exact mechanisms involved in vGPCR-induced angiogenesis and tumorigenesis are still being elucidated. vGPCR signaling induces the secretion of potent pro-angiogenic and pro-inflammatory chemokines and growth factors, such as VEGF, IL-8 and Gro-α that can promote inflammation, cell transformation, and angiogenesis through autocrine and paracrine mechanisms [6,7]. The large number of growth-promoting and survival pathways dysregulated by vGPCR could explain its potent transforming and oncogenic properties [8,9].

We previously reported that vGPCR activates expression of COX-2 that drives synthesis of PGE2, a proinflammatory molecule linked to tumor angiogenesis and upregulated in KS lesions [10]. Recent evidence indicates that PGE2 via signaling through its E2 receptor can activate β-catenin [11]. β-catenin is a dual function protein involved in the coordination of cell–cell adhesion and regulation of gene transcription. Numerous studies have identified aberrant activation of β-catenin signaling as a hallmark of many human cancers, which often result from stabilizing mutation in the genes encoding Wnt/β-catenin pathway components [12]. However, many cancers do not contain such mutations [13,14], suggesting that there are other factors that contribute to β-catenin activation. Indeed, Wnt/β-catenin signaling is commonly manipulated by oncogenic viruses. Hepatitis C (HCV) virus activates Wnt/β-catenin signaling, which appears to be key for the malignant transformation of hepatocytes seen in hepatocellular carcinoma [15]. Epstein Barr virus (EBV)-encoded protein, latent membrane protein 2A (LMP2A) causes β-catenin stabilization through activation of PI3K/Akt and inactivation of GSK-3β [16].

Here we explore the role of Wnt/β-catenin signaling in response to vGPCR, a lytic phase viral protein expressed in KS lesions. We show that expression of vGPCR in endothelial cells activates Wnt/β-catenin signaling and induces the expression of growth factors and cell-cycle regulators. The results from this study identify a novel molecular mechanism by which vGPCR modulates host signaling, which is essential to further understand the pathobiology of KS and identify novel therapeutic targets.

## Results and discussion

**Figure 1:**
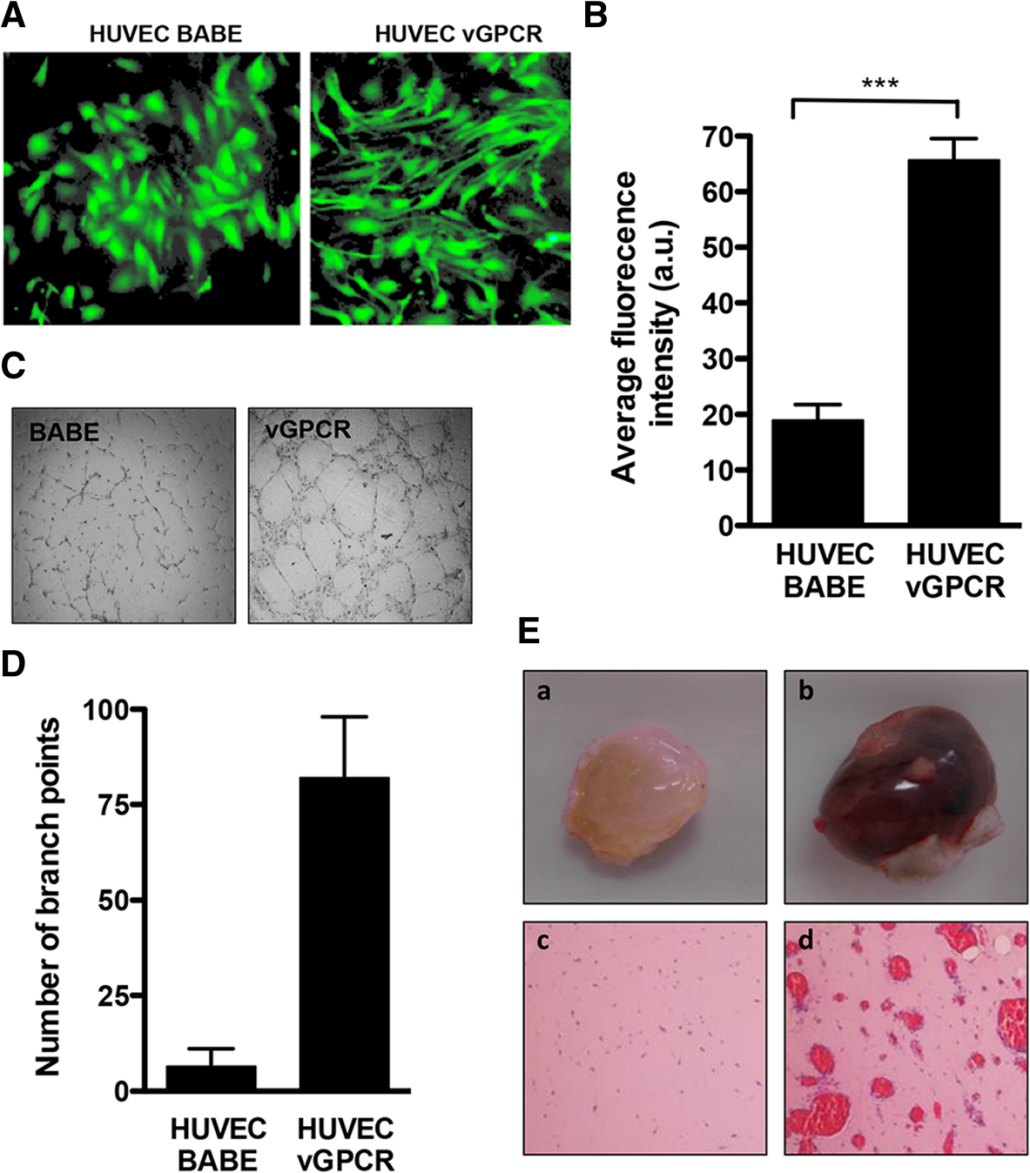
**Angiogenic and transforming properties of vGPCR. (A)** HUVECs were infected with the BABE retrovirus expressing vGPCR-GFP or a BABE-GFP control and imaged at 72 hr post transduction. **(B)** Cell migration was assayed using BD FluoroBlok cell culture inserts. BABE- and BABE-vGPCR-expressing HUVECs were loaded into the upper chamber of the inserts and allowed to migrate towards complete media for 12 hr. Migrated cells were stained with calcein AM and visualized by fluorescence microscopy. Average fluorescence intensity was determined using ImageJ software. Data are represented as the mean ± SEM (n = 3). **p < 0.001. **(C)** BABE- and BABE-vGPCR-expressing HUVECs were seeded in a 96-well plate pre-coated with growth factor-reduced Matrigel. Wells were examined for tubule-like structures after 6 hr and tube formation was quantified by counting the number of branch points **(D)**. Columns represent the average number of branch points from two independent experiments performed in duplicate ± SE. *p < 0.05. **(E)** NIH3T3 cells stably transduced with BABE-vGPCR or BABE were mixed with high concentration, growth-factor-reduced Matrigel. Matrigel-cell suspension was injected subcutaneously into the left and right flanks of 6–8 weeks old athymic NCr-nu/nu mice. n (BABE) = 4; n (vGPCR) = 6. Plugs were retrieved, fixed and processed for hematoxylin-eosin staining. Macroscopic appearance of representative plugs containing BABE- **(panel a)** or BABE-vGPCR-expressing cells **(panel b)**. Microscopic examination of hematoxylin-eosin stained sections from Matrigel plugs containing BABE- **(panel c)** or BABE-vGPCR-expressing cells **(panel d)**. vGPCR plugs show an increase in formation of blood vessels containing red blood cells.

Transgenic expression of vGPCR in mice is sufficient to induce cell proliferation, spindle cell morphology, angiogenesis and tumorigenesis that leads to formation of lesions similar to those in Kaposi’s sarcoma (6). As the spindle cells in KS lesions are believed to be of endothelial origin [17], primary human umbilical vein endothelial cells (HUVECs) were used to confirm the angiogenic properties of vGPCR. HUVECs transduced with a retrovirus expressing vGPCR exhibited spindle-like cell morphology that resembles that of endothelial cells found in KS lesions, while HUVECs transduced with the vector control (BABE) retained their cobblestone appearance (Figure 1A). Endothelial cell migration and re-organization into new vessels are essential steps of neoangiogenesis. vGPCR expression significantly enhanced migration of HUVECs as compared to the control cells (Figure 1B). In addition, vGPCR significantly increased the ability of HUVECs to form capillary, tube-like networks on a basement membrane (Figure 1C), which was quantified in (Figure 1D). Similarly, vGPCR-expressing NIH-3T3 fibroblasts injected subcutaneously into nude female mice displayed a marked increase in neovascularization and formation of luminal structures containing red blood cells (Figure 1E, panels b & d). The plugs containing the control cells appeared clear and without newly formed blood vessels or blood cells (Figure 1E, Panels a & c).

**Figure 2:**
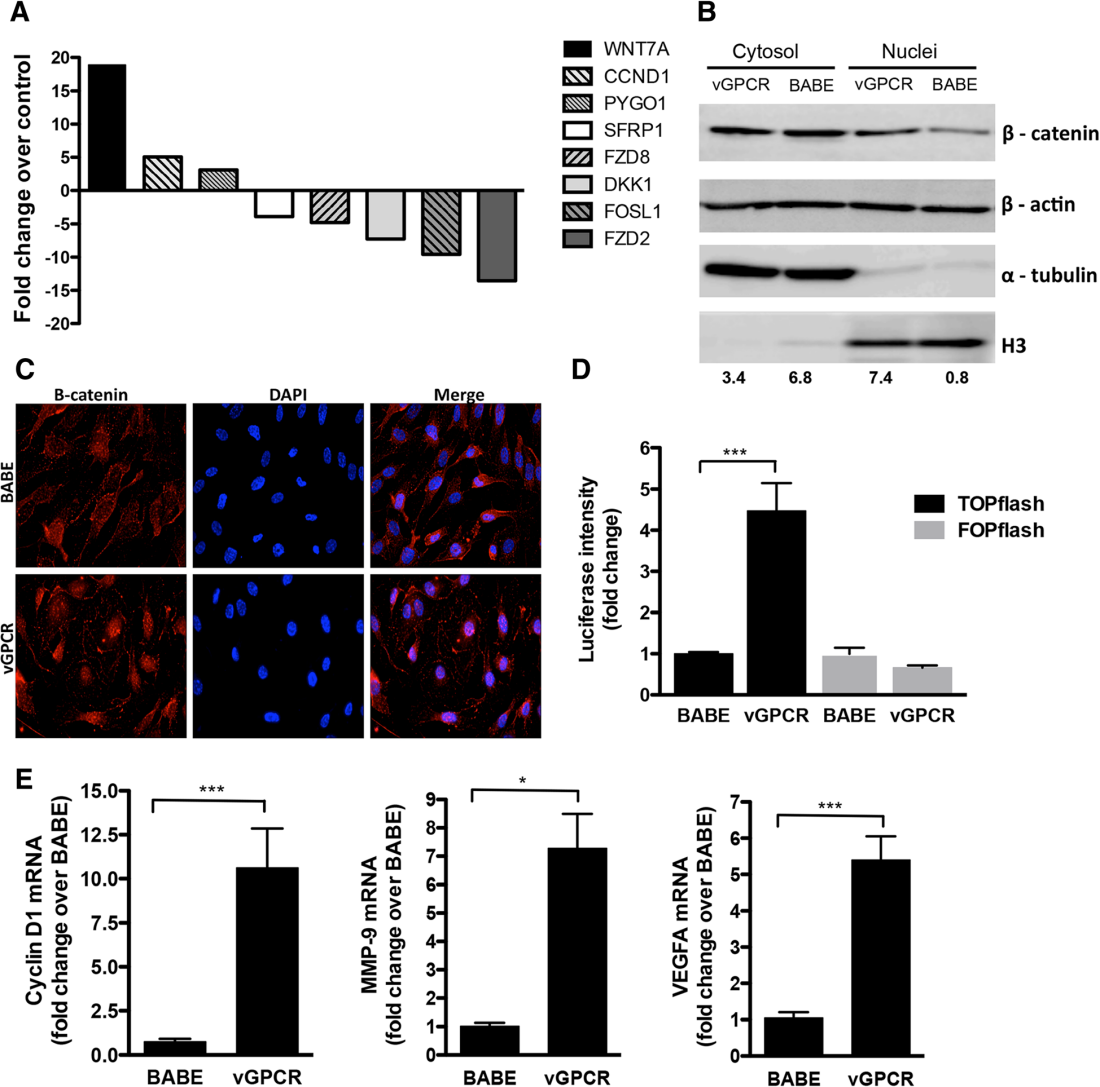
**vGPCR activates Wnt/β-catenin signaling in endothelial cells. (A)** Total RNA was isolated from BABE- and BABE-vGPCR-expressing HUVECs and analyzed with Human Wnt signaling pathway RT^2^ Profiler PCR array (SABiosciences, Frederick, MD). Genes that displayed greater than 3-fold change over control were considered significant. **(B)** HUVECs expressing vGPCR or BABE vector were serum starved for 16 hr and fractionated into cytosolic and nuclear components. Protein fractions were analyzed by Western blot with an anti-β-catenin antibody. Anti-γ-tubulin, and anti-H3 Western blots were included as controls for the purity of the fractionation procedure. β-actin served as a loading control. The densitometry data presented below the bands are arbitrary units normalized to the respective loading control (α-tubulin or histone H3). **(C)** HUVECs stably transduced with BABE or BABE-vGPCR were seeded on glass coverslips and stained with rabbit polyclonal antibody to β-catenin followed by AlexaFluor 555-conjugated anti-rabbit IgG. Nuclei were counterstained with DAPI. 40 X magnification. **(D)** HUVECs expressing vGPCR or control vector (BABE) were transiently transfected with TOPflash or FOPflash firefly luciferase-expressing plasmids. pRL-TK plasmid expressing *Renilla* luciferase was co-transfected as a control. Forty-eight hr after transfection, the cells were collected in lysis buffer and luciferase expression in cell lysates was measured and normalized to *Renilla* activity. Data are presented as the mean of results from two independent experiments each performed with triplicate transfections. ***p < 0.001. **(E)** Cyclin D1, MMP-9 and VEGFA mRNA levels in BABE-vGPCR- and BABE-expressing HUVECs were analyzed by qRT-PCR in triplicate. Each sample was normalized to the level of 36B4 expression. The data are presented as fold change (mean +/− SEM) relative to BABE control sample. *p < 0.05; ***p < 0.001.

Next, we investigated whether Wnt/β-catenin target genes and pathway components are altered by vGPCR. RNA from HUVECs expressing vGPCR or the control vector was analyzed using a Wnt Signaling Pathway RT^2^ Profiler PCR Array. Results indicated upregulation of several β-catenin target genes and ligands, including *cyclin D1*, *Wnt7A* and *pygopus 1* (*Pygo*) at least three-fold in vGPCR expressing cells compared to control (Figure 2A and Additional file 1). Cyclin D1 has a role in cell proliferation as an effector of G_1_–S progression [18]; Wnt7A is a canonical Wnt ligand that has been implicated to promote vascular growth and angiogenesis [19]; Pygo is a β-catenin binding protein required for the formation of transcriptional complexes [20]. The genes downregulated at least three-fold by vGPCR include the negative regulators of Wnt signaling secreted frizzled related protein 1 (SFRP1) and DKK1, as well as the Wnt receptors Frizzled 2 and 8. DKK1 and SFRP1 are often epigenetically inactivated in human cancers, including colorectal, cervical, prostate, and breast cancer [21,22]. Collectively, these results suggest that expression of vGPCR may activate Wnt signaling.

β-catenin acts as an intracellular transducer in canonical Wnt signaling. β-catenin is normally localized in the cytoplasm in an inactive state through its interaction with a multiprotein complex comprised of axin, adenomatous polyposis coli (APC) and two serine/threonine kinases, glycogen synthase kinase-3β (GSK-3β), and casein kinase 1 (CK1). Phosphorylation of β-catenin on serine-45 by CK1 followed by phosphorylation on serine-33 and −37 by GSK-3β marks β-catenin for polyubiquitination and proteasomal degradation. Wnt ligand binding to receptors on the surface of target cells initiates a cascade of events leading to disruption of the axin/APC/GSK-3β complex thus preventing phosphorylation and degradation of β-catenin. Accumulation of stable, hypophosphorylated β-catenin in the cytoplasm promotes its translocation to the nucleus (reviewed in [22]) where it binds T cell-specific factor(TCF)/lymphoid enhancer-binding factor-1 (LEF-1) DNA-binding factors to activate transcription of over fifty target genes involved in cell maintenance, proliferation and survival, such as *cyclin D1*, *c-myc*, VEGFA, *MMP-2* and *MMP-9* [22]. Thus, the subcellular distribution of β-catenin and its transcriptional activation upon vGPCR expression was next investigated. Levels of β-catenin were found to be higher in the nucleus of vGPCR-expressing cells than in the control cells, as determined by a Western blot analysis of nuclear and cytoplasmic fractions (Figure 2B). These observations were confirmed by immunofluorescence analysis, demonstrating that more β-catenin accumulates in the nucleus of vGPCR-expressing cells (Figure 2C).

**Figure 3:**
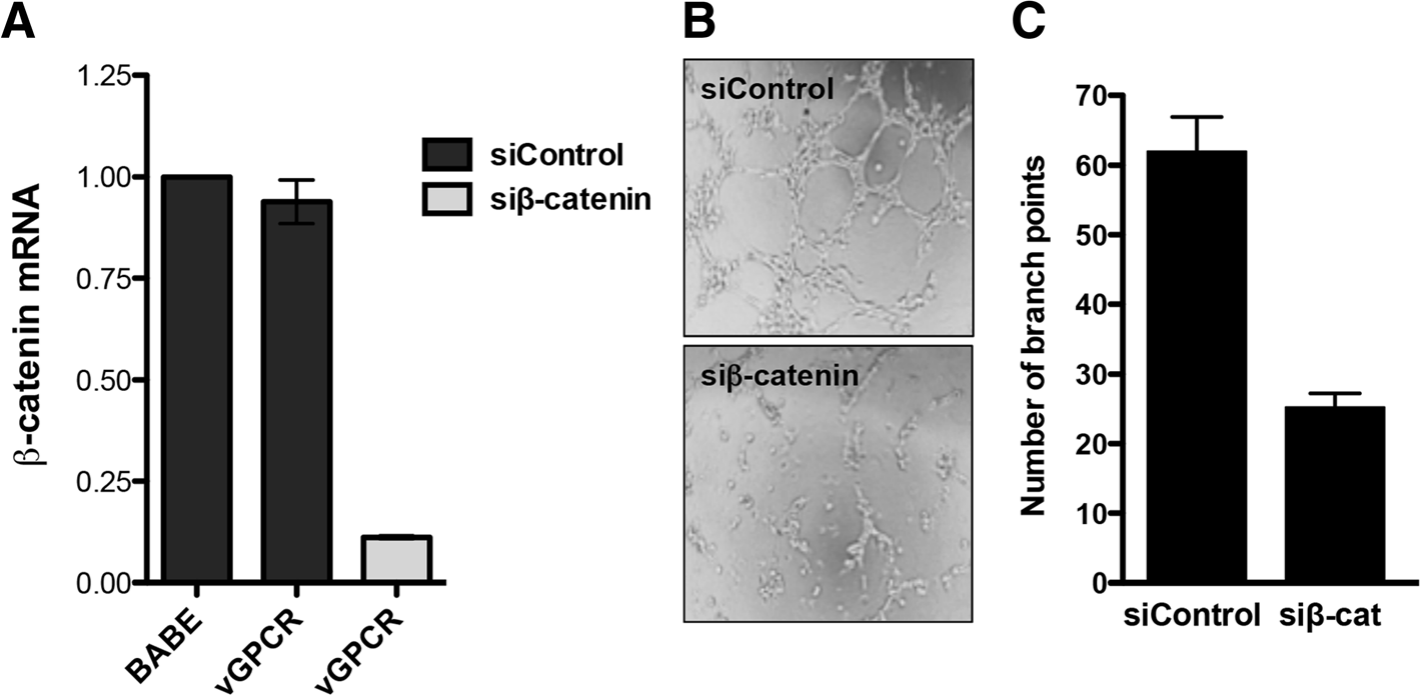
**Effect of β-catenin knockdown on tubule formation and β-catenin target gene expression.** HUVECs expressing vGPCR were transiently transfected with siRNA to β-catenin or with non-silencing siRNA control. **(A)** 72 hr following β-catenin silencing, knockdown efficiency was determined by qRT-PCR. β-catenin mRNA levels were normalized to 36B4 expression. **(B)** vGPCR-HUVECs were plated on Matrigel as described in Materials and Methods and allowed to form tubules. **(C)** The number of branch points was determined 6 hr after plating. The data is presented as an average of two independent experiments performed in duplicate. ***p < 0.001.

To confirm that vGPCR leads to accumulation of transcriptionally active β-catenin, HUVEC expressing vGPCR were transfected with a β-catenin/LEF1-dependent TopFlash reporter construct harboring three LEF/TCF binding sites upstream of the thymidine kinase minimal promoter, and 24 hr later, the luciferase activity of the reporter was measured. The TopFlash-specific promoter activity was significantly increased by vGPCR (Figure 2D). Consistent with these results, quantitative RT-PCR analysis indicated a significant increase in expression of β-catenin target genes *cyclin D1*, *MMP-9 and VEGFA* by vGPCR (Figure 2E). VEGFA, cyclin D1 and MMP-9 have been implicated in angiogenesis and KS development [23,24,25]. To substantiate the role of β-catenin in vGPCR-mediated angiogenesis, we utilized siRNA to decrease expression of β-catenin in cells stably expressing vGPCR (Figure 3A). Silencing β-catenin led to a decrease in endothelial tube formation, confirming a positive role of β-catenin in vGPCR-mediated angiogenesis (Figure 3B and C). However, β-catenin knockdown in vGPCR-expressing endothelial only slightly decreased the expression of the angiogenic factor cyclin D1 (data not shown). This may be due to residual β − catenin signaling activity in the knockdown cells, or the contribution of other signaling pathways that may also regulate expression of this gene. These results suggest that the increased angiogenic potential of vGPCR is mediated, at least in part, through activation of Wnt/β-catenin signaling. Further work is needed to determine if β-catenin knockdown will disrupt angiogenesis *in vivo*.

**Figure 4:**
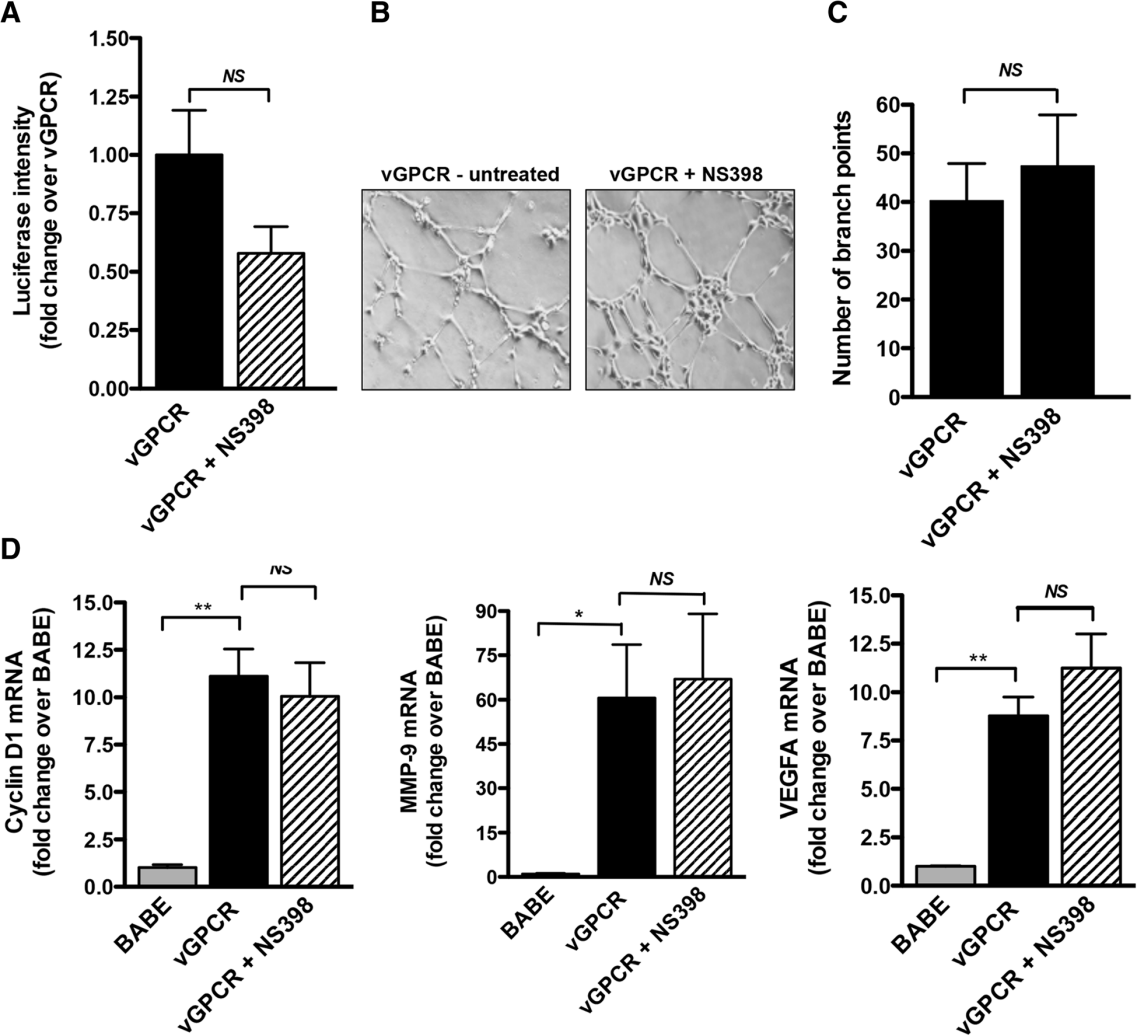
**Effect of COX-2 inhibition on Wnt/β-catenin activation by vGPCR. (A)** HUVECs expressing vGPCR were transiently transfected with TOPflash and pRL-TK plasmids. Twenty-four hr after transfection, the cells were treated with 100 μM of NS-398 or vehicle control for 24 hr. Cell lysates were collected and analyzed for luciferase expression. Data are presented as the mean from two independent experiments each performed in duplicate. *ns* – not significant. **(B)** HUVECS expressing vGPCR were pretreated with 100 μM NS-398 or vehicle control for 24 hr and seeded onto Matrigel-coated wells in medium supplemented with 100 μM NS398 or vehicle control for 6 hr. **(C)** The number of branch points was determined and expressed as the average from three replicate wells. *ns* – not significant. **(D)** Cyclin D1, VEGFA and MMP-9 mRNA expression was measured by qRT-PCR in control (BABE), vGPCR- expressing cells, and vGPCR-expressing cells that were treated with 100 μM NS-398 for 24 hr. The data are presented as fold change (mean +/− SEM) relative to control (BABE) sample. *p < 0.05; **p < 0.01; *ns* – not significant.

Activation of Wnt/β-catenin signaling by GPCRs has been previously demonstrated. There is evidence that the receptors for Wnt ligands, the Frizzled receptors, are heterotrimeric GPCRs [26,27]. In addition, classical GPCR proteins like the PGE2 receptor on colon cancer cells and the α-andrenergic receptors on cardiomyocytes, have been shown to activate canonical β-catenin signaling in a Wnt-independent manner (see review [28]). We next explored the contribution of COX-2 to Wnt/β-catenin activation by vGPCR. vGPCR-expressing HUVECs were transfected with the β-catenin reporter TOPflash, treated with the selective COX-2 inhibitor NS-398, and analyzed for luciferase expression. Inhibition of COX-2 with NS-398 failed to inhibit vGPCR-mediated activation of TOPflash, as there was no detected difference in activation of the reporter between treated and untreated cells (Figure 4A). Likewise, inhibition of COX-2 by NS-398 was also not sufficient to inhibit vGPCR-induced tube formation (Figure 4B and C), and the elevated expression of β-catenin target genes, *cyclin D1*, *MMP-9* and *VEGFA*, was unaffected by COX-2 inhibition (Figure 3D). Collectively, these results suggest that activation of Wnt/β-catenin by vGPCR occurs through a COX-2-independent mechanism.

**Figure 5:**
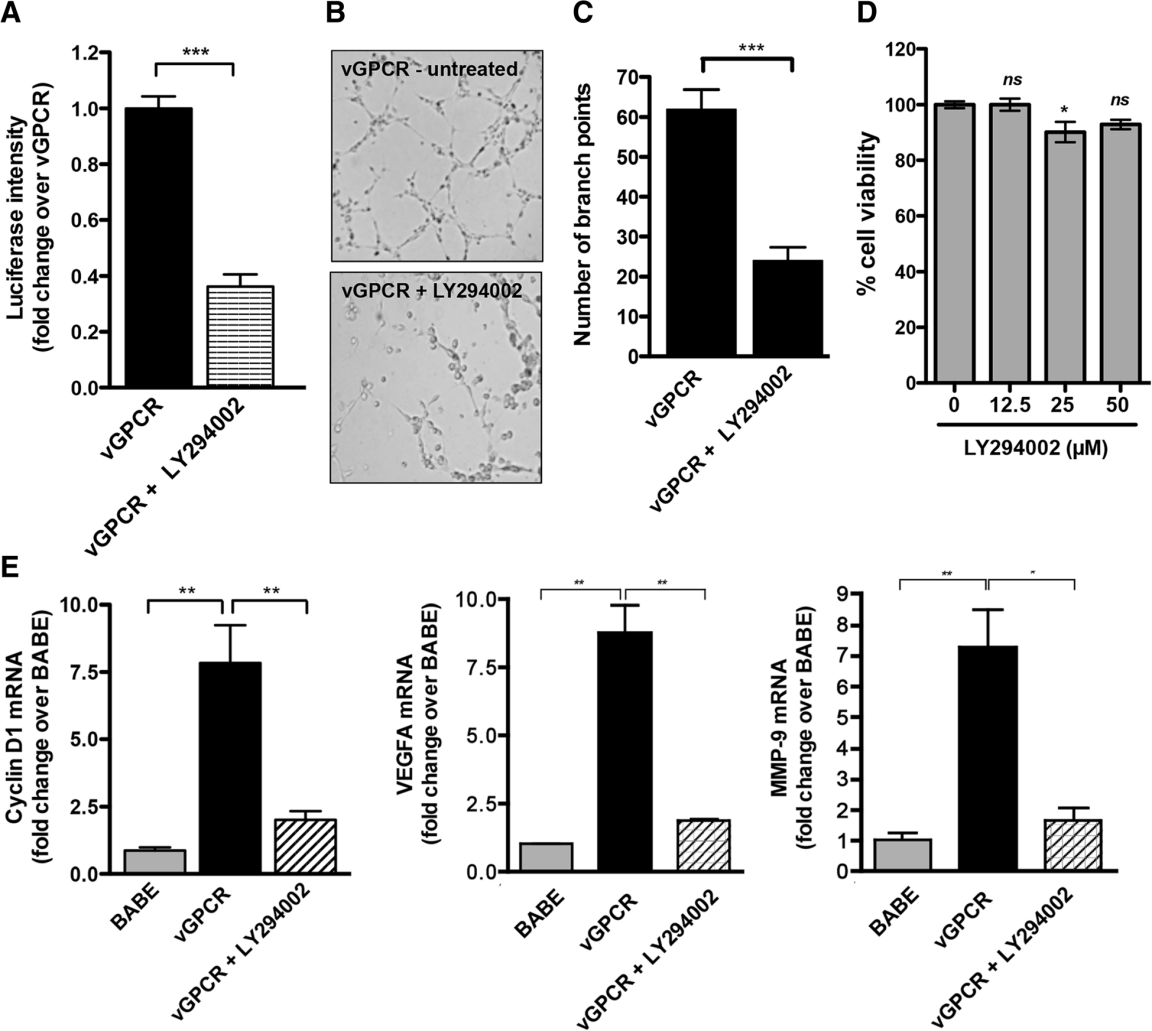
**Effect of PI3K inhibition on Wnt/β-catenin activation by vGPCR. (A)** HUVECs expressing vGPCR were transiently transfected with TOPflash and pRL-TK plasmids. Twenty four hr after transfection, the cells were treated with 25 μM LY294002 or vehicle control for an additional 24 hr. Cell lysates were collected and analyzed for luciferase expression as described in Figure 3A. **(B)** HUVECs expressing vGPCR were pretreated with 25 μM LY294002 or vehicle control for 12 hr and plated in 96-well plate coated with Matrigel in the presence of LY294002. **(C)** The number of branch points was determined as in Figure 3B. ***p < 0.001. **(D)** Cytotoxicity of increasing doses of LY294002 was determined by MTT assay *p < 0.05. **(E)** Cyclin D1, VEGFA and MMP-9 mRNA expression was measured by qRT-PCR in control (BABE), vGPCR- expressing cells, and vGPCR-expressing cells that were treated with 50 μM LY294002 for 24 hr. The data are presented as fold change (mean +/− SEM) relative to control (BABE) sample. *p < 0.05; **p < 0.01.

Activation of the PI3K/Akt signaling axis appears to be fundamental in vGPCR oncogenesis [29,30]. A report by Martin et al. [29] demonstrates that an isoform of PI3K, PI3Kγ, is necessary for vGPCR sarcomagenesis, as its inhibition causes tumor regression. Upon activation, PI3K activates its downstream effector Akt/protein kinase B (Akt) by promoting its phosphorylation at residues Ser 473 and Thr 308. Activated Akt promotes endothelial cell migration and differentiation through phosphorylating a wide range of downstream substrates. One downstream target of Akt is GSK-3β, which undergoes auto-inhibition when phosphorylated. In the absence of phosphorylation by GSK-3β, β-catenin cannot be targeted for degradation and is thus stabilized promoting its accumulation and subsequent nuclear translocation. Another study showed that activated PI3K/Akt can phosphorylate adherence junctions-associated β-catenin on S552, which causes β–catenin to dissociate from the junctions and translocate to the nucleus [31]. Skurk et al. reported that β-catenin–enhanced endothelial cell differentiation and migration are inhibited by PI3K/Akt inhibition, suggesting that Wnt/β-catenin promotes angiogenesis and blood vessel growth via the induction of PI3-K/Akt signaling in endothelial cells [32]. Therefore, the effect of PI3K inhibition on vGPCR-driven activation of β-catenin signaling was investigated next. TOPflash activity in vGPCR-expressing cells was significantly decreased following PI3K inhibition with LY294002 (Figure 5A). This was not due to increased cytotoxicity, as the compound resulted in a minimal reduction in cell viability (Figure 5D). In addition, inhibition of PI3K reduced the formation of capillary endothelial tubes (Figure 5B and C) and the expression of β-catenin target genes (Figure 5E), suggesting involvement of PI3K in vGPCR-induced activation of the β-catenin pathway. We plan to further investigate if β-catenin activation by PI3K/Akt occurs through AKT-mediated phosphorylation of GSK-3β or through direct phosphorylation of β-catenin by AKT. Further understanding of the cross talk between Wnt/β-catenin signaling and PI3K/Akt pathways and the role they play in vGPCR pathogenesis will help further elucidate the key steps in the angiogenic process and may help establish Wnt/β-catenin and PI3K pathways as novel therapeutic targets for treatment of KS.

## Methods

### Cell lines and retrovirus infection

Primary human umbilical vein endothelial cells (HUVECs) (Lonza, Allendale, NJ) were grown in M-199 media supplemented with 20% fetal bovine serum (FBS), 1% Penicillin/Streptomycin and 50 μg/mL ECGS (BD Biosciences, San Jose, CA) on vessels coated with 2% gelatin. Recombinant retroviral vectors expressing either GFP alone (BABE) or GFP and KSHV ORF74 (BABE-vGPCR) [10] were used to infect HUVECs at 40-60% confluency in complete media supplemented with 8 μg/mL polybrene (hexamethidrine bromide) (Sigma). GFP fluorescence was used to determine transduction efficiency between 48–72 hr post-infection. For all experiments, transduction efficiency was >80%.

### Quantitative real-time RT-PCR (qRT-PCR)

Total RNA was harvested from cells using the Qiagen *RNeasy* Kit (Qiagen) according to the manufacturer’s instructions. Total RNA (500 ng) was reverse transcribed using iScript cDNA Synthesis kit (Bio-Rad) and used in a qPCR using SYBR Green supermix (Bio-Rad). Oligonucleotide primers (Integrated DNA Technologies) used are as follows: cyclin D1 forward (5′-CGCCCTCGGTGTCCTACTTC-3′), cyclin D1 reverse (5′-GACCTCCTCC-TCGCACTTCTG-3′); VEGFA forward (5′- GGCAGAAGGAGGAGGGACAGAATC -3′), VEGFA reverse (5′- CATTTACACGTCTGCGGATCTTGT -3′); MMP-9 forward (5′-AGACGGGTATCCCTTCGACG-3′), MMP-9 reverse (5′-AAACCGAGTTGGAA-CCACGAC-3′); DKK1 forward (5′-TTCCAACGCTATCAACCTGC-3′), DKK1 reverse (5′- CAAGGTGGTTCTTCTGGAATACC-3′); c-myc forward (5′- GCCACGTCTCCA-CACATCAG-3), c-myc reverse (5′-TCTTGGCAGGAGGATAGTCCTT-3′); 36B4 forward (5′-TGGAGACGGATTACACCTTC-3′), 36B4 reverse (5′-CTTCCTTGGCTT-CAACCTTAG-3′); Relative quantitation was determined using the comparative C_T_ method with data normalized to 36B4 and calibrated to the average ΔC_T_ of BABE control at the specific time points.

### Western blotting

Cells were lysed in RIPA buffer (25 mM TrisHCl pH 7.6, 150 mM NaCl, 1% NP-40, 1% sodium deoxycholate, 0.1% SDS) supplemented with protease inhibitors. Protein samples were separated by SDS-PAGE and transferred to a nitrocellulose membrane. Membranes were blocked in 5% non-fat dry milk and incubated in primary antibody overnight at 4°C, followed by horseradish peroxidase-conjugated anti-mouse or anti-rabbit secondary antibodies (Invitrogen). Antibody-protein complexes were detected with ECL Plus (Amersham Biosciences) and exposed to chemiluminescent film. Primary antibodies: mouse anti-β-catenin (Santa Cruz Biotechnology; 1:500 dilution), mouse anti-β-actin (Abcam; 1:5,000), mouse anti-γ-tubulin (Abcam; 1:1000), anti-Histone 3 (Cell Signaling; 1:1000). Densitometric analysis was performed using ImageJ software (National Institutes of Health, Bethesda, MD).

### Luciferase analyses

HUVECs were transiently transfected with the TCF/LEF-1 reporter plasmid TOPflash, which contains multimeric TCF/LEF-1 sequences upstream of a firefly luciferase reporter gene. FOPflash plasmid containing mutated TCF/LEF-1 binding sites was used as a specificity control for TOPflash. Plasmids were purchased from Millipore (Bedford, MA). A Renilla luciferase-expressing plasmid pRL-TK (Promega) was co-transfected as an internal control. Cells were transfected with 1 μg of either TOPflash or FOPflash DNA and 0.1 μg of Renilla plasmid using the Neon transfection system (Invitrogen) according to the manufacturer’s instructions. Twenty-four hr after transfection, luciferase activity was assayed with a dual-luciferase reporter assay kit (Promega) and measured in a luminometer (Berthold Technologies). For each sample, the firefly luminescence signal was normalized to the corresponding Renilla signal.

### Small interfering RNA (siRNA) transfection

Cells were transfected with 100 nM siRNA to β-catenin (Santa Cruz) or the same concentration of non-silencing siRNA (Santa Cruz) using the NEON transfection system (Invitrogen). Twenty-four hours later cells were serum starved for 24 hr and harvested for subsequent assays.

### Isolation of cytoplasmic and nuclear fractions

Cytoplasmic and nuclear fractions were isolated from cells using a nuclear extract kit (Active Motif, Carlsad, CA) according to the manufacturer’s protocol. Resulting fractions were separated on SDS-PAGE and immunoblotted for β-catenin, H3, and γ-tubulin as described above.

### Immunofluorescence

Cells were seeded onto glass coverslips coated with 0.2% gelatin, and infected with BABE or BABE-vGPCR as described above. At indicated time points after infection, cells were fixed in 2% paraformaldehyde (Ted Pella) and permeablized in 0.1% Triton X-100. The cells were blocked in 5% BSA for 1 hr at room temperature and incubated with primary antibodies for 1 hr at room temperature followed by incubation with AlexaFluor-conjugated secondary antibodies and DAPI. Coverslips were mounted with ProLong gold antifade (Invitrogen) and imaged at 40X using a Zeiss Axioplan II microscope (Carl Zeiss).

### Migration assays

Cells were loaded into 8 μm FluoroBlok 24-well multiwell insert system (BD Biosciences) at 2x10^4^ cells per insert, in 200 μl of M-199 supplemented with 0.5% FBS. The bottom chamber of the insert was loaded with 800 μl of complete M-199. The cells were incubated at 37°C in 5% CO_2_ and allowed to migrate for 12 hr. The inserts were then labeled with calcein AM (Molecular Probes) and visualized by fluorescent microscopy. Three random fields from each insert were captured with a Nikon TE200 inverted fluorescent microscope (Nikon Instruments) and the average fluorescence intensity was determined by ImageJ software.

### Endothelial tube formation assay

Cells were trypsinized and resuspended in 100 μl of low serum (0.5%) M-199 medium without ECGS. Cells were seeded in a 96-well plate coated with 100 μl/well of growth factor reduced Matrigel (BD Bioscience) at a density of 1x10^4^ cells/well and incubated at 37°C for 6 hr. Tube formation was observed using a phase-contrast microscope. The number of tubes was quantified by counting the number of tube branch points.

### In vivo Matrigel plug angiogenesis assay

Animal experiments were approved by the Institutional Animal Care and Use Committee (IACUC) at Tulane University. NIH3T3 cells were stably transduced with BABE-vGPCR or BABE as control. For each cell population, 2 × 10^6^ cells were harvested in 200 μl of phenol-free, serum-free media containing 10 units/ml of heparin, and mixed with 200 μl of high concentration, growth-factor-reduced Matrigel (BD Bioscience). 400 μl of the Matrigel-cell suspension was injected subcutaneously into the left and right flanks (2 sites per mouse) of anaesthetized, 6–8 weeks old nu/nu athymic mice. Plugs were retrieved 7 days after implantation and fixed in 10% formalin overnight. After fixation, plugs were rinsed in PBS, paraffin embedded sectioned and stained with hematoxylin-eosin.

### Statistical analysis

Significant differences between experimental groups were determined by Student’s t-test or one-way analysis of variance (ANOVA) followed by Tukey’s post hoc *t* test using GraphPad Prism 4 software. Data are presented as the means ± standard error of the means (SEM).

### Reagents

The PI3K inhibitor LY294002 was obtained from Cell Signaling; the selective COX-2 inhibitor NS-398 was purchased from Cayman Chemicals. The concentration for each inhibitor is specified in the figure legends.

## Additional file

Additional file 1:
**Wnt Signaling Pathway RT** ^2^ **Profiler PCR Array results for KSHV infected HUVEC.**
