# Supplementary material for: Kaposi’s sarcoma-associated herpesvirus G-protein coupled receptor activates the canonical Wnt/β-catenin signaling pathway
Source: Virol J. 2014 Dec 17;11:218. doi: 10.1186/s12985-014-0218-8 (PMC4304609; doi:10.1186/s12985-014-0218-8)
Supplement: Additional file 1: — Wnt Signaling Pathway RT 2 Profiler PCR Array results for KSHV infected HUVEC. [file 12985_2014_218_MOESM1_ESM.docx]

Supplemental Table 1. Wnt Signaling Pathway RT^2^ Profiler PCR Array results for KSHV infected HUVEC. The PCR arrays consisted of 84 genes related to WNT-mediated signal transduction, 5 reference genes, 3 reverse transcription controls and 3 positive controls. Genes up-regulated in KSHV infected HUVEC compared to mock-infected HUVEC by at least 3 fold are in red and genes down-regulated 3 fold or more are in green.

| **Gene symbol** | **Description** | **Fold up- or down-Regulation** |
| --- | --- | --- |
|  |  | **KSHV infected/Mock infected** |
| AES | Amino-terminal enhancer of split | 1.18 |
| APC | Adenomatous polyposis coli | -1.12 |
| AXIN1 | Axin 1 | 1.36 |
| BCL9 | B-cell CLL/lymphoma 9 | -1.04 |
| BTRC | Beta-transducin repeat containing | 1.79 |
| FZD5 | Frizzled homolog 5 (Drosophila) | 1.10 |
| CCND1 | Cyclin D1 | 5.06 |
| CCND2 | Cyclin D2 | 1.45 |
| CCND3 | Cyclin D3 | 1.45 |
| CSNK1A1 | Casein kinase 1, alpha 1 | 2.06 |
| CSNK1D | Casein kinase 1, delta | -1.12 |
| CSNK1G1 | Casein kinase 1, gamma 1 | -1.47 |
| CSNK2A1 | Casein kinase 2, alpha 1 polypeptide | 1.10 |
| CTBP1 | c-terminal binding protein 1 | -1.20 |
| CTBP2 | c-terminal binding protein 2 | -1.38 |
| CTNNB1 | Catenin (cadherin-associated protein), beta 1, 88 kDa | -1.20 |
| CTNNBIP1 | Catenin, beta interacting protein 1 | -1.47 |
| CXXC4 | CXXC finger 4 | 2.20 |
| DAAM1 | Disheveled associated activator of morphogenesis 1 | -1.47 |
| DIXDC1 | DIX domain containing 1 | -1.69 |
| DKK1 | Dickkopf homolog 1 (Xenopus laevis) | -7.26 |
| DVL1 | Disheveled, dsh homolog 1 (Drosophila) | 2.06 |
| DVL2 | Disheveled, dsh homolog 2 (Drosophila) | -1.58 |
| EP300 | E1A binding protein p300 | -1.04 |
| FBXW11 | F-box and WD repeat domain containing 11 | 1.79 |
| FBXW2 | V-box and WD repeat domain containing 2 | 1.27 |
| FGF4 | Fibroblast growth factor 4 | 1.03 |
| FOSL1 | FOS-like antigen 1 | -9.58 |
| FOXN1 | Forkhead box N1 | 2.71 |
| FRAT1 | Frequently rearranged in advanced T-cell lymphomas | -2.23 |
| FRZB | Frizzled-related protein | 1.27 |
| FSHB | Follicle stimulating hormone, beta polypeptide | 1.03 |
| FZD1 | Frizzled homolog 1 (Drosophila) | -1.20 |
| FZD2 | Frizzled homolog 2 (Drosophila) | -13.55 |
| FZD3 | Frizzled homolog 3 (Drosophila) | -1.12 |
| FZD4 | Frizzled homolog 4 (Drosophila) | 1.67 |
| FZD6 | Frizzled homolog 6 (Drosophila) | 2.91 |
| FZD7 | Frizzled homolog 7 (Drosophila) | -2.95 |
| FZD8 | Frizzled homolog 8 (Drosophila) | -4.79 |
| GSK3A | Glycogen synthase kinase 3 alpha | 1.18 |
| GSK3B | Glycogen synthase kinase 3 beta | 1.67 |
| JUN | Jun oncogene | 1.27 |
| KREMEN1 | Kringle containing transmembrane protein1 | 1.03 |
| LEF1 | Lymphoid enhancer-binding factor 1 | 1.03 |
| LRP5 | Low density lipoprotein receptor-related protein 5 | -2.23 |
| LRP6 | Low density lipoprotein receptor-related protein 6 | -1.82 |
| MYC | V-myc myelocytomatosis viral oncogene homolog | -1.47 |
| NKD1 | Naked cuticle homolog 1 (Drosophila) | 1.03 |
| NLK | Nemo-like kinase | 1.03 |
| PITX2 | Paired-like homeodomain 2 | 1.67 |
| PORCN | Porcupine homolog (Drosophila) | 1.10 |
| PPP2CA | Protein phosphatase 2 (formerly 2A), catalytic subunit, alpha isoform | -1.58 |
| PPP2R1A | Protein phosphatase 2 (formerly 2A), regulatory subunit, alpha isoform | 1.45 |
| PYGO1 | Pygopus homolog 1 (Drosophila) | 3.12 |
| RHOU | Ras homolog gene family, member U | 2.20 |
| SENP2 | SUMO1/sentrin/SMT3 specific peptidase 2 | 1.56 |
| SFRP1 | Secreted frizzled-related protein 1 | -3.89 |
| SFRP4 | Secreted frizzled-related protein 4 | 1.03 |
| FBXW4 | f-box and WD repeat domain containing 4 | -1.04 |
| SLC9A3R1 | Solute carrier family 9 (sodium/hydrogen exchanger), member 3 regulator 1 | 1.10 |
| SOX17 | SRY (sex determining region Y)-box 17 | -1.47 |
| T | T, brachyury homolog (mouse) | -1.47 |
| TCF7 | Transcription factor 7 (T-cell specific, HMG-box) | 1.03 |
| TCF7L1 | Transcription factor 7-like (T-cell specific, HMG-box) | -1.47 |
| TLE1 | Transducin-like enhancer of split 1 (E(sp1) homolog, Drosophila) | -2.95 |
| TLE2 | Transducin-like enhancer of split 2 (E(sp1) homolog, Drosophila) | -1.82 |
| WIF1 | WNT inhibitory factor 1 | 1.03 |
| WISP1 | WNT1 inducible signaling pathway protein 1 | 1.03 |
| WNT1 | Wingless-type MMTV integration site family, member 1 | 1.03 |
| WNT10A | Wingless-type MMTV integration site family, member 10A | 1.03 |
| WNT11 | Wingless-type MMTV integration site family, member 11 | 1.03 |
| WNT16 | Wingless-type MMTV integration site family, member 16 | -1.95 |
| WNT2 | Wingless-type MMTV integration site family, member 2 | 1.03 |
| WNT2B | Wingless-type MMTV integration site family, member 2B | -1.04 |
| WNT3 | Wingless-type MMTV integration site family, member 3 | -1.95 |
| WNT3A | Wingless-type MMTV integration site family, member 3A | 1.92 |
| WNT4 | Wingless-type MMTV integration site family, member 4 | -1.69 |
| WNT5A | Wingless-type MMTV integration site family, member 5A | 1.92 |
| WNT5B | Wingless-type MMTV integration site family, member 5B | 1.03 |
| WNT6 | Wingless-type MMTV integration site family, member 6 | 1.03 |
| WNT7A | Wingless-type MMTV integration site family, member 7A | 18.90 |
| WNT7B | Wingless-type MMTV integration site family, member 7B | 1.03 |
| WNT8A | Wingless-type MMTV integration site family, member 8A | 1.03 |
| WNT9A | Wingless-type MMTV integration site family, member 9A | -2.08 |
| B2M | Beta-2 microglobulin | 2.20 |
| HPRT1 | Hypoxanthine phosphoribosyltransferase 1 | -1.58 |
| RPL13A | Ribosomal protein L13a | -2.08 |
| GAPDH | Glyceraldeyde-3-phosphate dehydrogenase | 1.27 |
| ACTB | Actin, beta | 1.18 |
